# Supplementary material for: Proteomic Analyses Reveal the Role of Alpha-2-Macroglobulin in Canine Osteosarcoma Cell Migration
Source: Int J Mol Sci. 2024 Apr 3;25(7):3989. doi: 10.3390/ijms25073989 (PMC11011979; doi:10.3390/ijms25073989)
Supplement: Supplementary file 1 [file ijms-25-03989-s001.zip › ijms-2893830-supplementary.pdf]

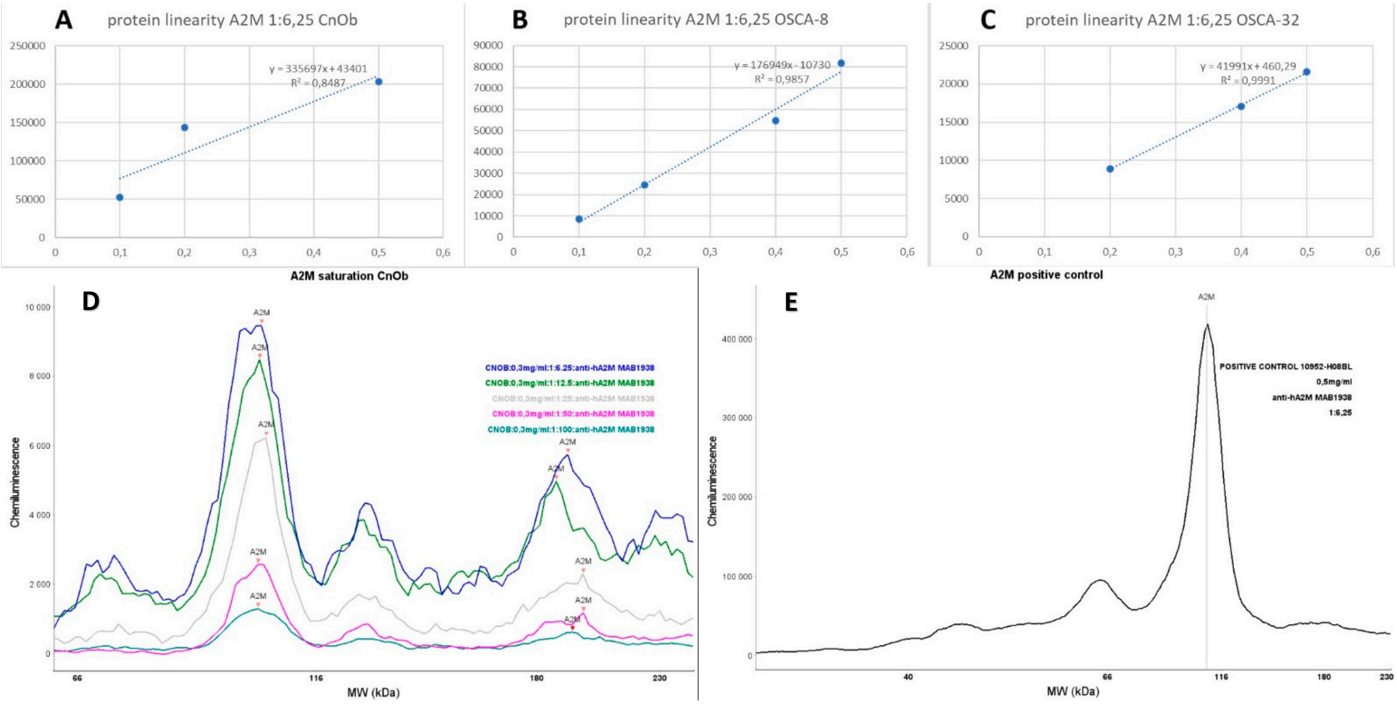

Figure S1. A2M antibody MAB1938 (Bio-Techne,Minneapolis,Minnesota,USA) validation for simple western technique for canine osteosarcoma cell lines (OSCA-8 and OSCA-32) and canine osteoblasts (CnOb). Antibody linear range for CnOb (A), OSCA-8 (B), OSCA-32 (C). Antibody saturation (D) , positive control with Recombinant Human alpha-2-macroglobulin Protein 10952-H08B (Sino Biological,Beijing,China) (E)
